# HER2 status of bone marrow micrometastasis and their corresponding primary tumours in a pilot study of 27 cases: a possible tool for anti-HER2 therapy management?

**DOI:** 10.1038/sj.bjc.6603584

**Published:** 2007-01-30

**Authors:** A Vincent-Salomon, J-Y Pierga, J Couturier, C D d'Enghien, C Nos, B Sigal-Zafrani, M Lae, P Fréneaux, V Diéras, J-P Thiéry, X Sastre-Garau

**Affiliations:** 1 Department of Tumor Biology, Institut Curie, Paris, France; 2 Department of Medical Oncology, Institut Curie, Paris, France; 3 Department of Surgery, Institut Curie, Paris, France; 4 Department of Translational Research, Institut Curie, 26 rue d'ulm 75248 Paris, Cedex 05, France

**Keywords:** breast cancer, HER2, bone marrow, micrometastasis, trastuzumab

## Abstract

Discrepancies have been reported between HER2 status in primary breast cancer and micrometastatic cells in bone marrow. The aim of this study was to assess HER2 gene status in micrometastatic cells in bone marrow and corresponding primary tumour. Micrometastatic cells were detected in bone marrow aspirations in a prospective series of 27 breast cancer patients by immunocytochemistry (pancytokeratin antibody). HER2 status of micrometastatic cells was assessed by fluorescence *in situ* hybridisation (FISH), respectively in 24 out of 27. Primary tumour HER2 status was assessed by immunohistochemistry (CB11 antibody) and by FISH in 20 out of 27 of the cases. HER2 was amplified or overexpressed in five out of 27 (18.5%) primary tumours and in four out of 27 (15%) micrometastatic cells. In two cases, HER2 was overexpressed and amplified in primary tumour, but not in micrometastatic cells, whereas, in one case, HER2 presented a low amplification rate (six copies) in micrometastatic cells not found in the primary tumour. We demonstrated that negative and positive HER2 status remained, in the majority of the cases, stable between the bone marrow micrometastasis and the primary tumour. Therefore, the efficiency of anti-HER2 adjuvant therapy could be evaluated, in a clinical trial, by sequential detection of HER2-positive micrometastatic cells within the bone marrow, before and after treatment.

HER2 overexpression, observed in 15–30% of breast cancers, is associated with a poor outcome, especially in node-positive breast carcinoma (Gusterson *et al*, 1992; Perou *et al*, 2000). HER2 status remains stable between the primary tumour site and distant metastasis (Niehans *et al*, 1993; Gancberg *et al*, 2002; Vincent-Salomon *et al*, 2002; Carlsson *et al*, 2004) or regional lymph node metastasis (Simon *et al*, 2001). In contrast, HER2 has been found to be overexpressed in 60–100% in bone marrow micrometastatic cells, independently of the primary tumour status (Braun *et al*, 2001b). Cytotoxic agents currently used for chemotherapy in high-risk breast cancer patients do not completely eliminate micrometastatic cells in bone marrow (Braun *et al*, 2000a) and bone marrow micrometastasis in breast cancer patients is associated with a poor outcome (Braun *et al*, 2000b, 2005; Naume *et al*, 2004). In this context, a targeted therapy, specific for micrometastatic cells, would be appropriate. However a recent study showed HER2 heterogeneous overexpression in bone marrow micrometastatic cells could be detected in patients with HER2-negative primary tumours. This heterogeneity may reduce the efficacy of an immunotherapy-based strategy in an adjuvant setting (Solomayer *et al*, 2006). The aim of this pilot study was to assess *HER2* gene status by fluorescence *in situ* hybridisation (FISH) in micrometastatic cells in bone marrow of breast cancer patients and to compare it to *HER2* primary tumour status, in order to evaluate if anti-HER2 therapy in adjuvant setting, could be given to patients after an assessment of HER2 status of the primary tumour only. In addition, as the cytokeratin antigens detected in epithelial micrometastatic cells are not specific to cancer cells, morphological analysis of positive detected cells is a major step in the identification of micrometastatic cells. In this perspective, the second aim of our study was to confirm that the isolated cytokeratin-positive (CK+) cells detected in bone marrow aspirates and interpreted as micrometastasis actually corresponded to tumour cells. We therefore documented the neoplastic nature of the cells by assessing the gene status of other frequently amplified oncogenes in breast carcinomas (Taccagni *et al*, 1997; Al-Kuraya *et al*, 2004; Orsetti *et al*, 2004), especially *CCND1* (cyclinD1) and *MYC*.

## MATERIALS AND METHODS

### Patients

Cytokeratin positive micrometastatic cells were detected in bone marrow aspirates in a prospective series of breast cancer patients. Bone marrow samples positive for micrometastatic cells and the corresponding primary tumours were obtained from 27 patients (three stage II, one stage III, one local relapse and 22 stage IV). A single bone marrow aspiration was performed under local anaesthesia from the posterior iliac crest before chemotherapy in an adjuvant setting or for metastatic disease. Informed consent was obtained from all patients.

### Bone marrow specimens

Techniques have been described previously (Pierga *et al*, 2004). Briefly, 3–5 ml of bone marrow aspirate was collected on EDTA (Vacutainer, Becton Dickinson, Le Pont de Claix, France). Components of the bone marrow aspirate from the two iliac crests were processed under sterile conditions. Each sample was diluted by addition of half the volume of Hanks solution (Gibco BRL, Invitrogen, Cergy, Pontoise, France). Samples were separated by Ficoll/Hypaque density centrifugation (Sigma, St Louis, MO, USA; density, 1.077 g ml^−1^) in Leucosep tubes (Polylabo, Au Verney, Servion, France) (830 g, 15 min, 20°C). The mononuclear cells (MNCs) layer was harvested from each tube, combined, diluted in 50 ml of Hanks and centrifuged at 360 g, 5 min at 20°C. Cells were resuspended in PBS/0.1% bovine serum albumin (BSA). After dilution to 3% in pure acetic acid for red cell lysis, an aliquot of the cell suspension was counted. The MNCs were resuspended in PBS/BSA at 1.10^6^ ml^−1^. One millilitre of the cell suspension was cytocentrifuged twice onto polylysine-coated slides at 580 *g* for 3 min (Hettich Universal 16A cytocentrifuge). The supernatant was carefully removed from each slide after the first cytocentrifugation and the slides were allowed to dry in air overnight. Slides were stored at −20°C and then at −80°C until staining.

### Immunocytochemical staining

The pancytokeratin (CK) monoclonal antibody A45-B/B3 (Micromet, Munich, Germany and Chromavision, San Juan, Capistrano, USA), which recognises several cytokeratin epitopes which characterise CK 8, CK 18 and CK 19, was applied for cell detection (Stigbrand *et al*, 1998). The immunostaining procedure was standarised by using an automated device (Cadenza, Shandon, France). Before staining, cytospots were fixed with 4% paraformaldehyde for 5 min, and then dried for 15 min at room temperature. Endogenous alkaline phosphatase was then blocked with TBS solution (Sigma) with 2% human AB serum, for 15 min. This solution was used to dilute primary and secondary antibodies. After blocking, the slides were incubated with the primary antibody A45 B/B3 (2 *μ*g ml^−1^ for 40 min). Control slides were incubated under the same conditions with a mouse monoclonal anti-FITC IgG1 (1/1250) (Sigma). Slides were incubated for 20 min with secondary polyclonal rabbit anti-mouse antibody (Dako France, Trappes, France, A/S, Glostrup, Denmark). After each step, the slides were rinsed for 5 min in TBS 1 X solution. Immune complexes were revealed by the alkaline phosphatase-anti-alkaline phosphatase (APAAP) technique (Dako) (1/50) for 25 min. The chromogen reaction was performed for 20 min with a colorimetric substrate of fuchsin solution (2.5% in 2 N HCl) (New Fuchsin, Sigma) with 4% NaNO_2_, 8% *β*-naphthol (Sigma) and 2% levamisole (Dako). Cells were counterstained with Mayer hematoxylin (1 min) (Sigma) diluted to one out of three in distilled water. The specimen was then rinsed under running water for 5 min and then in TBS. Slides were coverslipped using Faramount mounting medium (Dako). Mononuclear cells (3 × 10^6^) in three slides were evaluated for each patient. Negative controls, stained with anti-FITC monoclonal mouse antibody, were performed on an equivalent number of cells (i.e. three slides, 3 × 10^6^ mononuclear cells) for each patient.

Positive controls were obtained with bone marrow from ‘normal’ donors undergoing orthopedic surgery, spiked with SKBR3 or MCF7 cell lines, 10–10^2^ for 10^6^ mononuclear cells per cytospot. One positive control slide and one negative control slide were added to each series of 20 stained slides in the automated device.

### CK+ cell detection

Cell detection was performed by manual screening with an optical microscope. Criteria for evaluation of immunostained cells in bone marrow were adapted from Borgen *et al* (1999) based on the results of the European ISHAGE Working Group for standarisation of tumour cell detection. The main criteria were a large cell size, a high nuclei/cytoplasm ratio and the absence of obvious haematopoietic cell morphology.

### *HER2* status

*HER2* status of micrometastatic cells in BM was assessed by FISH on slides on which CK+ cells had been detected. Slides were rinsed in PBS, then treated by pepsin (0.05% in 0.01 N HCl), for 5 min at 37°C, then dehydrated in ethanol series. Digoxigenin-labelled *HER2* probe solution (Zymed Laboratories Inc., South San Francisco, CA, USA) was laid onto the slides, which were covered by coverslips. In some cases, directly SpectrumOrange-labelled *Cyclin D1* or myc probes (Vysis, Downers Grove, IL, USA) were added to the mix in order to increase the probability of detecting abnormal micrometastatic cells. Simultaneous denaturation of the probes and cell DNA was performed at 75°C for 2 min. Slides were then incubated overnight at 37°C in humid chamber, for hybridisation. Rinsing was performed in 0.4 × SSC/0.3% Igepal at 75°C, for 4 min, then in the same solution at 20°C, for 2 min, followed by 5 min in PBS. *HER2* hybrids were revealed by incubation with a FITC-labeled anti-digoxigenin antibody (Roche Diagnostics, Basel, Switzerland), 1/100 dilution, for 30 min, at 37°C. Finally, slides were mounted in Vectashield/DAPI (Vector Laboratories, Burlingame, CA, USA). Preparations were analysed by microscope and when technically possible, all CK+ cells detected by their cytoplasmic fluorescence were photographed under FITC, and, in the case of *Cyclin D1* or myc hybridisation, under SpectrumOrange excitations.

HER2 status of primary tumour was assessed by immunohistochemistry (CB11 antibody, Novocastra, Newcastle, UK) and by FISH in 20 cases out of the 27 cases, with available and suitable blocks for *HER2* FISH analysis.

HER2 immunostainings and FISH were performed on histological tissue sections prepared from a representative sample of the primary tumour. Immunohistochemical procedures for the analysis of HER2 expression were defined to provide a strong correlation between HER2 overexpression and gene amplification status, as determined by FISH (Couturier *et al*, 2000). After rehydration and antigenic retrieval in citrate buffer (10 mM, pH 6.1), tissue sections were incubated with the CB11 anti-p185 ^HER/neu^ monoclonal antibody (Novocastra, Newcastle UK), for 1 h, at 1/800 dilution. Staining was revealed with the Vectastain Elite ABC peroxidase mouse IgG kit (Vector Burlingame, CA, USA), using diaminobenzidine (Dako A/S, Glostrup, Denmark) as chromogen. Under these conditions, normal epithelial cells were not immunostained and therefore constituted an internal negative control.

Immunostainings were scored as strong, weak or negative according to the percentage of labelled tumour cells and membrane staining intensity. Cases were considered to be positive when at least 60% of cells were stained (Bilous *et al*, 2003; Vincent-Salomon *et al*, 2003). HER2 status was then classified as overexpressed (strong or moderate staining) or not significantly overexpressed.

Fluorescence *in situ* hybridisation was performed according to the same protocol as that already described earlier for micrometastasis. In addition, after deparaffinisation, slides were first treated with a protein digesting enzyme, at 37°C, for 10 min.

Cytokeratins 8/18 expression was also assessed on these 20 out of 27 primary tumours according to a previously published protocol (Azoulay *et al*, 2005).

## RESULTS

Patient characteristics are summarised in Table 1. The mean age was 55.6 years (range: 36–75 years). Tumours were invasive ductal carcinomas in 85% of cases with a histological grade II or III in 14/25 (56%).

A visceral metastasis was observed 22 of the cases (22/27 cases, 81%). Nineteen cases presented bone metastases (19/27, 70% of cases). Eight of 22 stage IV patients had synchronous metastasis at primary diagnosis. Patients with metastatic disease received chemotherapy as first-line treatment in 65% of cases (15 patients), or second-line treatment (four patients)(17.5%) or third-line treatment (four patients) (17.5%).

*HER2* status in primary tumours and bone marrow micrometastasis are summarised in Table 2. 6/27 (22.2%) primary tumours had HER2 overexpression (2+ and 3+). In five cases, the intensity of staining was strong (3+) and observed in 100% of tumour cells of the invasive component. In 4/4 (100%) cases assessable for FISH analysis, this overexpression was associated to *HER2* amplification. The remaining case with moderate (++) staining did not show any amplification by FISH. Therefore, 5/27 (18.5%) tumours presented *HER2* amplification and overexpression.

Micrometastatic cells in bone marrow were observed in all selected cases. The cell morphology on cytospots was interpreted according to the ISHAGE criteria for tumour cells. The cells were large, with a high nucleus/cytoplasm ratio. In 19 out of 27 cases, the micrometastatic cells formed clusters. The number of micrometastatic cells ranged from 1 to 1500 per slide examined.

In four out of 27 (15%) (95%CI: 2–28%) cases *HER2* amplification was observed in bone marrow micrometastatic cells. When more than 20 cells were observed and interpretable the amplification was homogeneous. In two cases, *HER2* was amplified and overexpressed in the primary tumour, but not in distant BM micrometastatic cells and in one case, *HER2* presented a very low level of amplification in BM micrometastatic cells (six copies) and not in the primary tumour (two copies). At least, only two (50%) of four cases with *HER-2* amplification in primary tumours showed *HER-2* amplification in micrometastatic cells, and three (75%) of four cases with *HER-2* amplification in micrometastatic cells showed *HER-2* amplification in the primary tumours.

**Figure 1:**
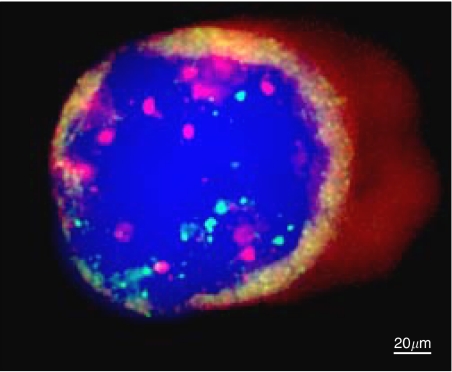
One micrometastatic cell CK+ (intracytoplasmic red labelling) with *HER 2* amplification (red spots) and *CCND1* amplification (green spots).

*CCND1* gene status was assessed in 20/27 of the cases. *CCND1* was amplified in seven of these 20 cases (35%). Notably, four of these seven *CCND1* amplified cases showed also an amplification of *HER2* (Figure 1). In the remaining three cases, this amplification confirmed that the detected *HER2*-negative CK+ cells actually corresponded to tumour cells.

*MYC* gene status was assessed in six cases and was amplified in one case, in which HER2 and CCND1 were also amplified (Table 2).

**Figure 2:**
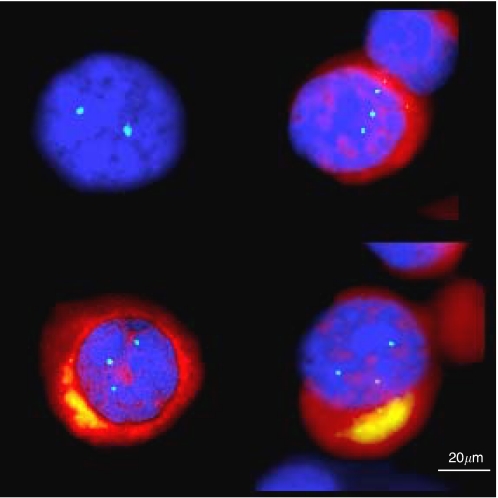
Chromosome 17 trisomy (blue spots) in CK+ cells (cytoplasmic red labelling) and disomy (blue spots) in cytokeratine-negative cell.

Four cases, without *HER2* amplification, presented three *HER2* gene copies per nucleus and thus demonstrated a *HER2* overrepresentation in relation with a chromosome 17 trisomy. The presence of chromosome 17 trisomy was another proof of malignancy of these micrometastatic cells (Figure 2) (Table 2).

All analysed cases showed cytokeratin 8/18 expression in their primary breast tumour, ranging from 10 to 100% of positive cells per case.

## DISCUSSION

In this pilot study, we wanted to document the stability of the *HER2* status between primary tumours and their bone marrow micrometastasis. We observed that the majority of the HER2-negative tumours were associated with HER2-negative micrometastasis except in one case in which micrometastatic cells demonstrated a lower level of *HER2* amplification. In addition, in our series of breast carcinomas, 15% of bone marrow micrometastatic cells presented *HER2* amplification. This rate is very close to that observed in primary breast tumours. *HER2* gene amplification appears thus to occur before bone marrow micrometastatic process in breast cancer and to remain stable during bone marrow micrometastatic spread.

This result is in accordance with those concerning visceral metastases and local and regional metastases (Barnes *et al*, 1988; Niehans *et al*, 1993; Masood and Bui, 2000; Simon *et al*, 2001; Gancberg *et al*, 2002; Vincent-Salomon *et al*, 2002; Carlsson *et al*, 2004). Recently, in a meta-analysis of the published data concerning HER2 status stability among primaries and metastases, Carlsson *et al* (2004) confirmed that there was no drastic modification in HER2 status between primary tumours and their locoregional lymph node metastases and their distant visceral metastases.

Our results and these published data on the stability of HER2 status between primary and metastatic tumours are in contrast with the recently published studies by Schmidt-Kittler *et al* (2003) and by Klein *et al* (Mercapide *et al* 2002) showing that micrometastatic cells demonstrated fewer chromosomal alterations, such as losses and gains detected by single-cell CHG analysis, than primary tumour cells (Mercapide *et al* 2002; Schmidt-Kittler *et al*, 2003). Another recently published study also showed that *HER2* amplification was more frequently observed in circulating cells than in primary tumours and therefore concluded that *HER2* amplification could be acquired during the metastatic process (Meng *et al*, 2004). The HER2 amplicon corresponds to a 280-kb minimal region of amplification at the HER2 locus of chromosome 17q arm, in breast cancer. The amplification is significantly associated with increased expression of six of the 10 genes located within this region (HER2, GRB7, PNMT, MLN64, MGC9753 and MGC 1483) as described by Kauraniemi *et al* (2003). This amplicon has been observed since the early stage of *in situ* carcinoma (Van de Vijver *et al*, 1988). The observation of *HER2* amplification exclusively in disseminated cells, suggests a selection of clones within the primary tumour that harboured initial *HER2* amplification and that were underrepresented in the primary tumour, for example, by cytotoxic agents, rather than an acquisition of this amplification *de novo* within the metastatic cells.

Previous studies by Pantel *et al* (1993), Braun *et al* (2001a) and Solomayer *et al* (2006), reported high rates of HER2 overexpression ranging from 60 to 100% of cases, analysed by immunocytochemistry only. These rates could therefore be explained by technical aspects, that is, excessively sensitive immunohistochemistry technique. Using the FISH approach, we demonstrated that primary breast tumours and bone marrow micrometastasis demonstrate the same range of *HER2* activation, ranging around 15% of the cases (Table 3).

The gene expression profile of metastasis and their primaries has also been compared and has been shown to cluster together (Ramaswamy *et al*, 2003; Weigelt *et al*, 2003). In the literature, *Cyclin D1* amplification in primary breast tumours ranged between 10 and 23%. In our series, the *CyclinD1* (CCND1) amplification rate was 35% (Al-Kuraya *et al*, 2004). This rate is therefore higher than previously reported for ductal carcinomas, although our series is very small to derive any conclusions about this issue. However, we can speculate that the amplification rate might be higher in this group of metastatic and advanced breast carcinomas. Amplification of *HER2* with other oncogenes has been reported previously, particularly in a recently published FISH study (Al-Kuraya *et al*, 2004). In this work, *HER2* was associated with *CCND1* amplification in 18% of cases. *CCND1* was amplified in 20% of cases. Determination of coamplification rates of major oncogenes such as *MYC* and *CCND1* in breast carcinomas should provide important information regarding prognosis. It has also been recently reported, during the 2005 San Antonio meeting, that *MYC* status could be a predictive parameter of tumour response to anti-HER2 therapy (Gianni *et al*, 2005).

In conclusion, the *HER2* status assessed by FISH in isolated micrometastatic cancer cells in bone marrow was well correlated with that of primary tumour. Our pilot study showed that in the majority of the cases, the stable, positive or negative, status of *HER2* during the bone marrow micrometastatic process. This observation on a small series of cases should be confirmed on a larger scale and identification of HER2-positive micrometastatic cells in breast carcinomas could constitute part of patient management in a future clinical trial, in order to select patients for anti-HER2 adjuvant therapy. Repeated assessment of the presence of micrometastases could also be part of the follow-up and evaluation of the efficacy of anti-HER2 therapy.

## Figures and Tables

**Table 1:** Patients and primary tumour characteristics

**Characteristics**	***N*=27**	**(%)**
*Age(years)*		
⩽50	8	30
>50	19	70
		
*Clinical stage*		
Stage I	0	0
Stage II	3	11
Stage III	1	4
Stage IV	22	81
Local relapse	1	4
		
*Tumour size (mm)*		
⩽20	5	18
] 20; 40]	8	30
>40	10	37
ND	4	15
		
*Histological grade*		
Grade I	5	18
Grade II	14	52
Grade III	6	22
ND	2	7
		
*Histological type*		
Ductal invasive	23	85
Lobular invasive	4	15
		
*Nodal status*		
0	8	30
1–3	2	7
⩾4	7	26
ND	10	37
		
*Hormonal status*		
ER+	16	59
PR+	9	34
ND	2	7
		
*Vascular invasion*		
+	13	48
−	6	22
ND	8	30

Abbreviation: ND=not determined.

**Table 2:** Descriptive results of HER2 status in primary tumours and in bone marrow by FISH and by immunohistochemistry

			**Oncogenes status in bone marrow**		
**HER2 status in primary tumour**			**HER2**	**CCND1**	**MYC**
**Cases**	**IHC (% positive cells)**	**No. of copies**	**No. of copies**	**No. of copies**	**No. of copies**
1	30	2 ×	2 ×	2 ×	ND
2	0	3 ×	3 ×	2 ×	ND
3	0	ND	3 ×	>20 ×	2 ×
4	100	12–15 ×	15 ×	12–15 ×	ND
5	0	2 ×	2 ×	ND	ND
6	0	2 ×	2 ×	2 ×	ND
7	0	ND	2 ×	2 ×	2 ×
8	0	3 ×	3 ×	ND	ND
9	0	2 ×	2 ×	2 ×	ND
10	0	2 ×	2 ×	2 ×	ND
11	0	2 ×	2 ×	2 ×	2 ×
12	0	ND	2 ×	12 ×	ND
13	0	ND	2 ×	2 ×	ND
14	0	2 ×	ND	ND	ND
15	0	2 ×	2 ×	2 ×	ND
16	0	2 ×	6 ×	>10 ×	ND
17	0	2 ×	ND	ND	ND
18	100	8–20 ×	20 ×	15 ×	1 ×
19	15	2 ×	2 ×	2 ×	ND
20	100	12 ×	2 ×	2 ×	ND
21	0	ND	3 ×	>10 ×	ND
22	0	2 ×	ND	ND	ND
23	60	2 ×	2 ×	2 ×	ND
24	100	8–20 ×	3 ×	ND	ND
25	100	ND	>20 ×	>20 ×	>10 ×
26	0	ND	2 ×	ND	ND
27	0	2 ×	2 ×	2 ×	2 ×

Abbreviations: ND=not determined; FISH: fluorescence *in situ* hybridization; IHC: Immunohistochemistry.

**Table 3:** Correlation between *HER2* status assessed by FISH in micrometastatic cells and by FISH and or immunohistochemistry in primary tumors

	**HER2 status in primary tumors (n=27)**	
**HER2 status in micrometastatic cells**	**Amplified/overexpressed**	**Not amplified**
Amplified	3	1
Not amplicited	2	21
Total	5	22

Abbreviation: FISH: fluorescence *in situ* hybridization; IHC: Immunohistochemistry.
